# Tuberculosis infection control measures in healthcare facilities in Moyen-Ogooué Province, Gabon

**DOI:** 10.1186/s12913-021-07236-z

**Published:** 2021-11-05

**Authors:** Anja Vigenschow, Bayodé Romeo Adegbite, Jean-Ronald Edoa, Abraham Alabi, Akim A. Adegnika, Martin P. Grobusch, Marguerite Massinga-Loembe

**Affiliations:** 1 Centre de Recherches Médicales de Lambaréné and African Partner Institution, German Center for Infection Research (CERMEL), Lambaréné, Gabon; 2 Institut für Tropenmedizin, Universität Tübingen and German Center for Infection Research, Tübingen, Germany; 3 Center of Tropical Medicine and Travel Medicine, Department of Infectious Diseases, Amsterdam Public Health, Amsterdam University Medical Centers, location Amsterdam, Amsterdam Infection & Immunity, University of Amsterdam, Amsterdam, The Netherlands; 4 Masanga Medical Research Unit, Masanga, Sierra Leone; 5 Institute of Infectious Diseases and Molecular Medicine, University of Cape Town, Cape Town, South Africa

**Keywords:** Tuberculosis, Infection control, Healthcare facility, Healthcare workers, Gabon

## Abstract

**Background:**

Healthcare workers (HCW) are at higher risk of tuberculosis (TB) than the general population. We assessed healthcare facilities for their TB infection control standards and priorities.

**Methods:**

A standardised tool was applied. The assessment was conducted by direct observation, documents review and interviews with the facility heads.

**Results:**

Twenty healthcare facilities were assessed; 17 dispensaries, an HIV-clinic, a private not-for-profit hospital and a public regional hospital. In both hospitals, outpatient departments, internal medicine wards, paediatric wards, emergency departments; and the MDR-TB unit of the public regional hospital were assessed. In Gabon, there are currently no national guidelines for TB infection control (TBIC) in healthcare settings. Consequently, none of the facilities had an infection control plan or TBIC focal point. In three departments of two facilities (2/20 facilities), TB patients and presumed TB cases were observed to be consistently provided with surgical masks. One structure reported to regularly test some of its personnel for TB. Consultation rooms were adequately ventilated in six primary care level facilities (6/17 dispensaries) and in none of the hospitals, due to the use of air conditioning. Adequate personal protective equipment was not provided regularly by the facilities and was only found to be supplied in the MDR-TB unit and one of the paediatric wards.

**Conclusions:**

In Moyen-Ogooué province, implementation of TBIC in healthcare settings is generally low. Consequently, HCW are not sufficiently protected and therefore at risk for *M. tuberculosis* infection. There is an urgent need for national TBIC guidelines and training of health workers to safeguard implementation.

**Supplementary Information:**

The online version contains supplementary material available at 10.1186/s12913-021-07236-z.

## Background

Due to possible occupational exposure to infectious TB patients, several studies have shown that healthcare workers (HCW) are at higher risk to acquire TB than the general population [1]. TB transmission in healthcare facilities does not only pose a risk for HCW, but also to fellow patients and visitors. Particularly in settings with a high HIV prevalence, the prevention of nosocomial transmission is crucial, as people living with HIV are highly susceptible to TB.

Although HCW are at the frontline of the fight against TB, the importance of infection control has long been undervalued. However, increasing evidence of nosocomial transmission of multi-drug resistant and extensively drug-resistant TB [2] has raised concern and awareness of the problem.

In settings where resources are limited, TB infection control (TBIC) can be challenging, as measures such as ultraviolet germicidal irradiation (UVGI) filters are expensive and difficult to maintain. However, the World Health Organization (WHO) recommends a number of TBIC measures for healthcare facilities that are feasible to be implemented in resource-limited settings [3].

According to the WHO, so-called managerial controls form the framework for any TBIC activities. They include the development of TBIC guidelines on a national level and the formation of a committee responsible for their implementation, as well as the provision of an appropriate budget and adequate human resources.

At facility level, there are administrative and environmental measures to be taken as well as personal protective equipment (PPE) to be put in place. Administrative TBIC measures focus on the triage of presumed TB cases, promotion of cough hygiene, and early diagnosis, as exposure mostly takes place when TB patients are not yet recognised as such. In addition, suggested measures include regular testing and surveillance of HCW.

While environmental measures aim to improve the ventilation of the facility and therefore reduce the number of infectious particles in the air, PPE in terms of particular respirators such as N95 masks may avoid the inhalation of these particles, when worn by a person exposed to an infectious TB patient. These measures can help to minimise the risk of nosocomial TB transmission as well as of other airborne diseases. Although TBIC recommendations are available for resource-limited settings, previous research has shown that their implementation is often insufficient. The problem is exacerbated by inadequate infrastructure or lack of HCW, resulting in patient overcrowding, delayed diagnosis, and consequently increased TB transmission. Data on the implementation of TBIC in sub-Saharan Africa is scarce. Studies from Uganda [4] and South Africa [5] reported that in participating health facilities where the survey was conducted, TBIC procedures were not well-applied. Lack of staff, space, and finances in the health-care system were identified as obstacles to TBIC implementation [4]. Furthermore, access to state-of-the-art molecular tools to identify transmission chains by sequencing are scarce, at least on institutional level and outside research contexts.

Gabon (population of 2.17 million in 2019) is one of three African countries, together with Lesotho (2.13 million) and South Africa (58.56 million), with an TB incidence >500/100,000 (578/100.000) [6]. The tuberculosis mortality rate was estimated by WHO as 110 per 100.000 population in 2019 [6]. The situation is aggravated by accumulating evidence of high drug resistance rates [7, 8] as well as an HIV prevalence of around 5 % [7, 9]. Routine data collected officially report on average two cases of TB among health care workers each year, but the basis of this report is unclear. At the time of this study, there was no systematic TB training programme for HCW implemented in Gabon [10].

 As there are no national TBIC guidelines available, we assumed that HCW as well as other patients in healthcare facilities in Moyen-Ogooué province are not sufficiently protected from TB transmission. The study was initiated to gain insight into current TBIC practices in different healthcare facilities in Moyen-Ogooué province, in order to properly quantify the dimension of the problem, with the intention to establish baseline data for the future implementation of TBIC measures.

## Methods

### Study design

We conducted a cross-sectional study to assess the status of TB infection control measures implementation in healthcare facilities in Moyen-Ogooué province, Gabon. This assessment aimed to contribute to the evidence base for the upcoming national TB infection control guidelines and to establish a follow-up and monitoring framework for implementation. In addition, we wanted to identify particular risk areas that need to be focused on as part of training and technical support activities.

### Study setting

**Fig. 1:**
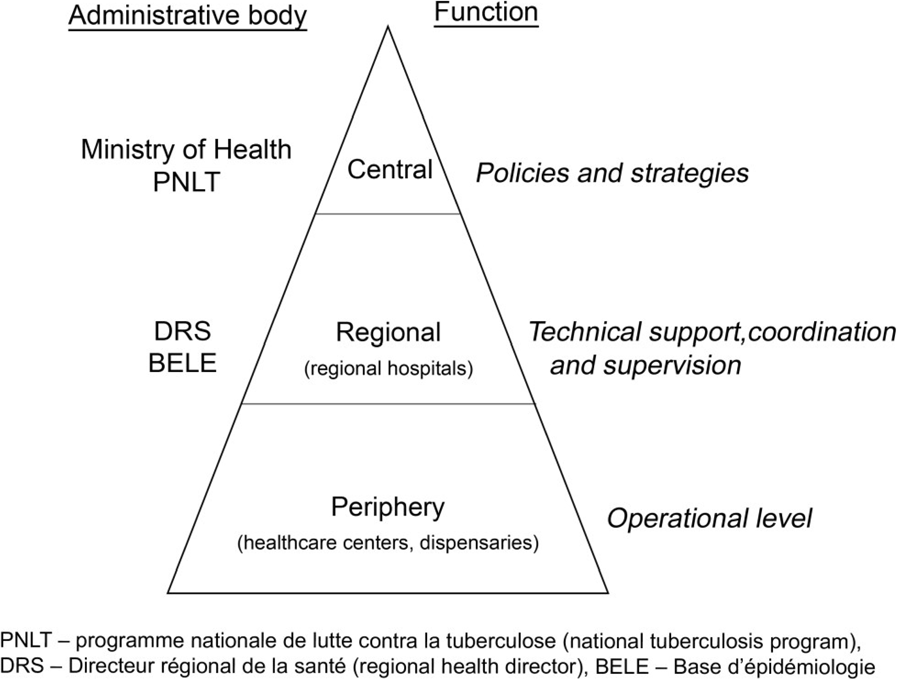
Healthcare level pyramid of Gabon

Gabon is an upper-middle-income country with considerable in-country inequity. The health infrastructure is characterised by a ratio of 25 hospital beds per 10,000 inhabitants, and a need for more health care workers and facilities at all levels. The preventive and promotion of heath action are not yet implemented in many health facilities. The management of tuberculosis is limited to few selected health facilities. The Gabonese healthcare administration consists of a tiered system composed of 10 sanitary regions, with the Libreville/Owendo sanitary region representing the central level. The study was conducted in the province of Moyen-Ogooué, representing the sanitary region ‘Centre’, with Lambaréné, a town with 30.000 inhabitants, as its capital city. Facilities in Lambaréné are considered as part of the intermediate level of the health care pyramid (Fig. 1).

In order to obtain a representative overview of TB infection control activities in Moyen-Ogooué province, we assessed all facilities representing all levels of the Gabonese healthcare system present in this region (peripheral and intermediate). Patients in Moyen-Ogooué typically seek healthcare in dispensaries first and are then referred to a larger facility in Lambaréné. Therefore, we considered the peripheral level facilities to be equally important as the larger intermediate level facilities with regard to TB infection control.

**Fig. 2:**
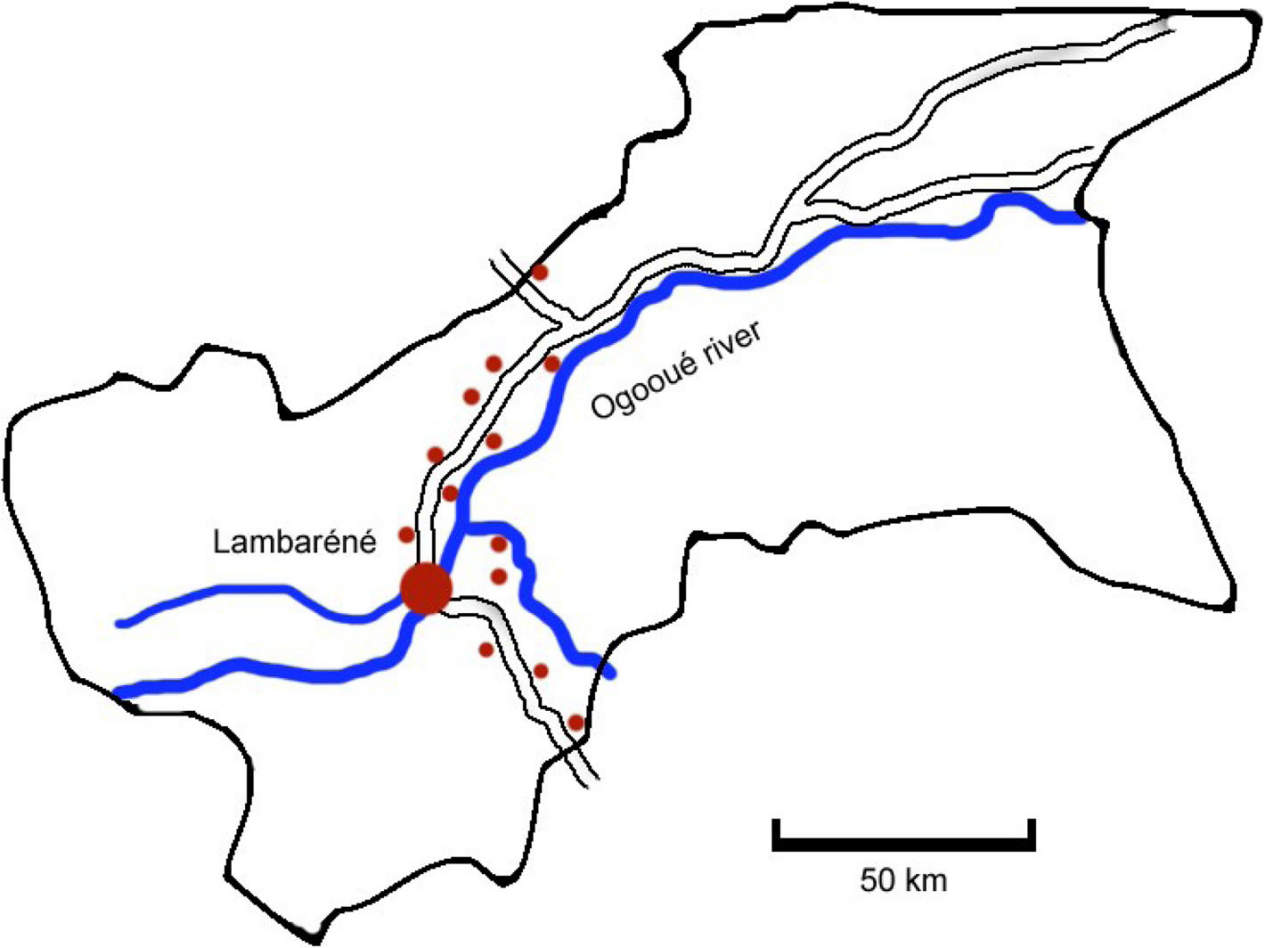
Sketch (not to scale) of healthcare facilities chosen for the study across Moyen Ogoué Province

 At the intermediate healthcare level, we included the two hospitals, the regional public reference hospital and a private non-profit hospital, as well as the ambulatory HIV-clinic (Centre de Traitement Ambulatoire, CTA). At the peripheral level, we included all four urban dispensaries located in Lambaréné, and rural dispensaries located along the main road axis of the province. These included 13 dispensaries located between the towns Tchad and Bifoun (Fig. 2). Hence, in total, all 20 healthcare facilities in Moyen-Ogooué province were assessed.

In both hospitals, departments considered to be particularly at risk for nosocomial TB transmission were assessed independently. The outpatient department (OPD) including the radiology unit, and the emergency departments (ED) were considered as the patients’ main hospital ports of entry. In addition, the internal medicine wards (IM), the pediatric wards (PD) and, in one of the hospitals, the newly set up MDR-TB-treatment unit (MT) were also assessed as commonly used for TB patient hospitalisation.

### Study period

The assessments were conducted between November 2016 and March 2017. In the major health facilities, each individual department was visited for two to three days. Dispensaries were assessed during single visits.

### Study procedures

A standardised assessment tool was developed, based on the WHO recommendations for implementation of TBIC in healthcare settings [3] and on the manual ‘Implementing TB infection control in health care facilities’ from Wits Reproductive Health and HIV Institute (WRHI)(Supplementary Material 1), and piloted in the framework of this study. This tool addressed various aspects of managerial, administrative and environmental controls as well as the use of personal protective equipment (PPE). A shortened version was used for the dispensaries. All assessments were carried out by the same investigator (AV). The following key information was collected at all sites; TBIC internal guidelines and committee notes, triage of coughing patients, patient education, screening of staff, facility structure and ventilation as well as PPE. Furthermore, available TB-related documentation, i.e. TB registers, patient files and training logs were reviewed. Any other relevant observation made during the assessment period was also documented.

The assessments were conducted by health care worker interviews and direct observations (non-participant observation) in order to provide a more objective assessment, as suggested elsewhere [4, 5, 11]. Staff were not informed of the goal of the direct observation. Implementation of infection control measures and practices were directly observed and recorded. In-depth interviews were conducted with the respective person in charge of infection control or, if such a person was not in place, the respective head of department. If certain procedures could not be assessed by direct observation during the assessment period, staff knowledge about the corresponding institutional policy or procedure was investigated during the interviews. If applicable, available documentation was reviewed to confirm answers provided during in the interview.

### Data management and statistical analysis

The data was then converted into colour-coded dashboards, representing the level of implementation for each infection control measure. Data for the dispensaries were collected in Excel tables, converted into colour charts. The proportions were calculated with SPSS Version 24.0 (IBM Corp., Armonk, New York, USA).

### Development of a new assessment tool

As the assessment tool was not optimal in this setting, we developed an adapted questionnaire at the end of this study based on the experiences made during this study, which can be used for future follow-up studies. The new tool focuses on specific factors that are of importance regarding the context and applicability to this setting. Furthermore, it contains distinct information on conducting the assessment and means of verification. The tool can be used to obtain comparable follow-up data on the baseline assessment of this study.

## Results

### Managerial controls

As there was absence of managerial controls on a national level, there were, consequently, no managerial controls implemented at facility level in Moyen-Ogooué province; neither in terms of setting performance standards, nor in measuring performance, or in having an action plan for taking corrective actions when necessary. None of the facilities had an infection control plan or TBIC focal point. Consequently, there was also no budget for TBIC. This was the first time that TBIC measures were systematically assessed in Gabon.

### Administrative controls

**Fig. 3:**
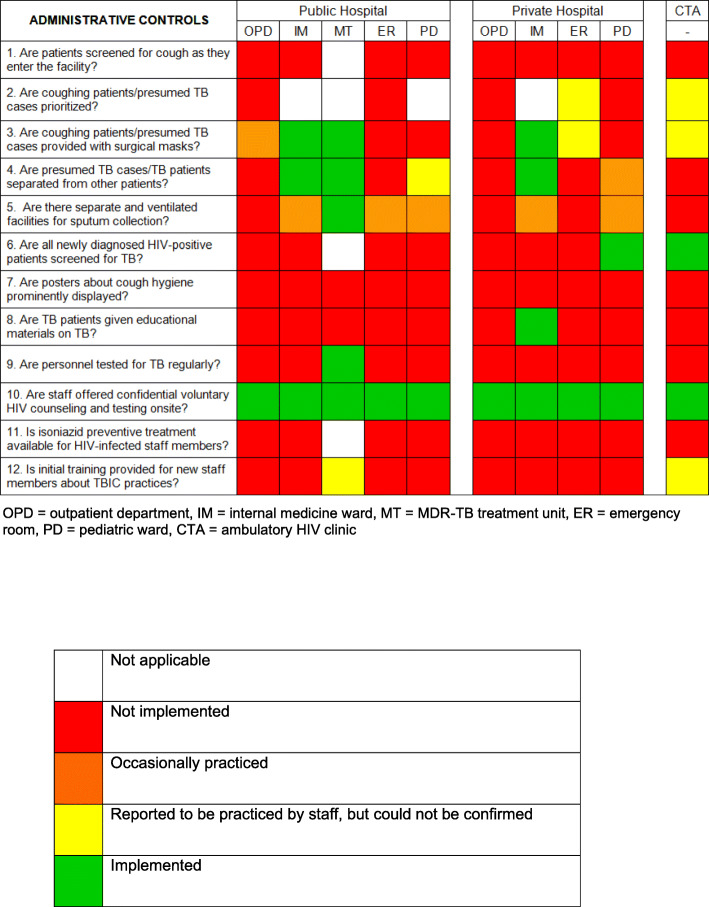
Implementation of administrative controls. Green colour indicates that the TBIC action was *Implemented*; Yellow colour indicates that the TBIC action were *reported to be practiced by staff, but could not be confirmed*; orange colour indicates that the TBIC *action were occasionally practiced*; red colour indicates that the TBIC action was *Not implemented*

Figure 3 presents the state of implementation of administrative TBIC measures in each individual department of the three intermediate-level healthcare facilities.

None of the facilities had implemented the systematic triage of coughing patients, as it is recommended by WHO [3].That notwithstanding, there were certain administrative TBIC measures that were consistently practiced in all three departments; mainly that coughing patients were provided with surgical masks and separated from other patients in the MT and the IM wards of both hospitals. These practices were reported to be in place in other departments as well. However, this information could not be confirmed by our observation due to a lack of TB cases during the assessment period.

Sputum collection was either performed at the patients’ home or, if a patient was hospitalised, in the isolation room. Only one department ensured that sputum collection was performed outside and away from other people, which minimises the risk of infecting others during this procedure.

Across the two hospitals, two out of 10 departments actively screened all newly diagnosed HIV-infected patients for TB.

There was an overall lack of patient education materials regarding TB and TBIC. None of the facilities had posters on cough hygiene, and educational material in terms of an informational leaflet for TB patients was available only in one department.

While all facilities provided free access to HIV-testing and counseling for staff, only one department screened its personnel regularly for TB. Isoniazide preventive therapy for HIV-infected HCW was generally unavailable.

In two departments, staff was reportedly trained in TBIC by the head physician and, regarding the MT, in collaboration with CERMEL(Centre de Recherches Médicales de Lambaréné). However, in both cases it could not be confirmed as there was no training documentation.

### Environmental controls

**Fig. 4:**
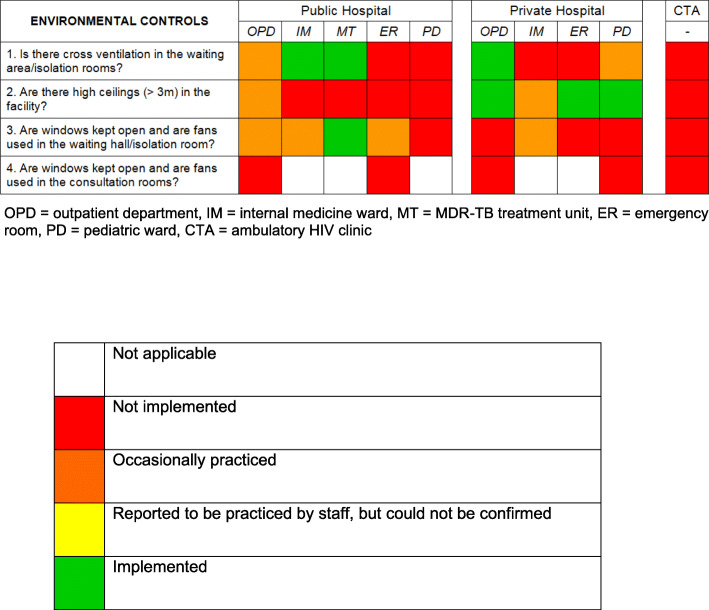
Implementation of environmental controls. Green colour indicates that the TBIC action was *Implemented*; orange colour indicates that the TBIC *action was occasionally practiced*, red colour indicates that the TBIC action was *Not implemented*

Environmental controls are depicted in Fig. 4. Each facility had several structural strengths and weaknesses regarding environmental TBIC. Four out of five individual departments in the private hospital had high ceilings. The spacious OPD (outpatient department) waiting areas were open to all sides, and were therefore considered as well-ventilated. There was cross-ventilation in the isolation rooms in the IM and MT of the public hospital. Other isolation rooms and/or waiting areas did not have optimal natural ventilation due to their architecture.

Open windows and the consistent use of fans were only found in the MT of the public hospital. In the other nine departments, these measures were only occasionally taken, rather than systematically implemented. None of the consultation rooms were adequately ventilated, as air conditioning (AC) was used and consequently, windows were closed.

### PPE

**Fig. 5:**
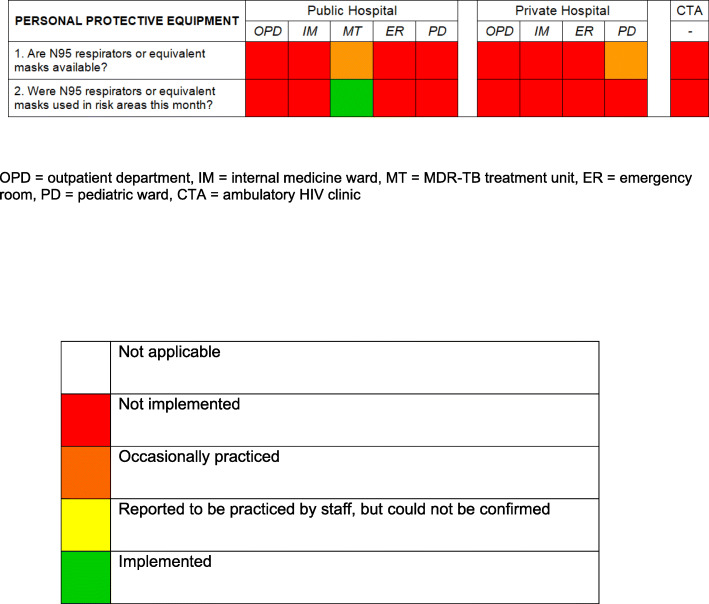
Implementation of personal protective equipment. Green colour indicates that the TBIC action was *Implemented*; orange colour indicates that the TBIC *action *was occasionally practiced; red colour indicates that the TBIC action was *Not implemented*

Availability and use of PPE are summarised in Fig. 5. None of the facilities provided adequate respirators for their staff. The MT was provided with FFP3 masks by the collaborating research institute. There were N95 masks occasionally available in the paediatric ward of one of the hospitals, but they were not observed to be used.

### Other findings

There were reports of active TB cases amongst HCW in both hospitals; one HCW fell ill from active TB during the assessments. However, these cases could not be verified, and due to a lack of routine sequencing facilities of clinical isolates, it remains likely but, in the end, unclear if they were due to nosocomial transmission.

The reviewing of TB registers and patient files revealed that documentation of TB cases and treatment was often incomplete, and numbers were inconsistent with the officially notified cases, which leads to the assumption that there is a need of strengthening the reporting and surveillance systems to gain more information about local TB epidemiology.

### Dispensaries

The baseline characteristics of the 17 dispensaries regarding the three months prior to the assessment are presented in Table 1. The total number of patients seeking consultation in those preceding three months ranged from 15 to 1253 patients across facilites. In most facilities, approximately a third of the patients were presenting with cough as one of their lead symptoms.

**Table 1:** Key characteristics of 17 dispensaries regarding staff/patient ratios during the three months preceding the survey carried out in 2016-2017)

		Total patients (n)	Coughing patients (n)	Proportion (%)	Number of staff^1^ (n)	Staff/patient ratio (%)
Dispensary		.	.	.	.	
*Urban*	Atsie	346	101	29.2	4	1
	Isaac	1253	238	19	5	0
	Magnang	210	44	21	6	3
	Moussamoukougou	206	61	29.6	1	-
*Rural*	Tchad	309	98	31.7	1	-^2^
	Kery Paga	288	47	16.3	1	-
	Nombakélé	74	7	9.5	2	3
	Zilé St Martin	22	6	27.3	1	5
	Siat Zilé	236	16	6.8	2	1
	Nzoghe Bang	31	9	29	2	6
	Kongoulé	44	13	29.5	1	2
	Adanhe	15	3	20	1	7
	Medang Nkoghe	15	6	40	1	7
	Nkoghe Mboum	28	10	35.7	1	4
	Benguie 2	30	3	10	1	3
	Benguie 4	65	24	36.9	1	2
	Paris-Bifoun	121	54	44.6	2	2

^1^staff including nurse and assistant nurse; ^2^inferior to 1 %

All rural dispensaries were managed by maximally two assistant nurses. Only three urban dispensaries employed qualified nurses holding post-graduate diplomas.

**Fig. 6:**
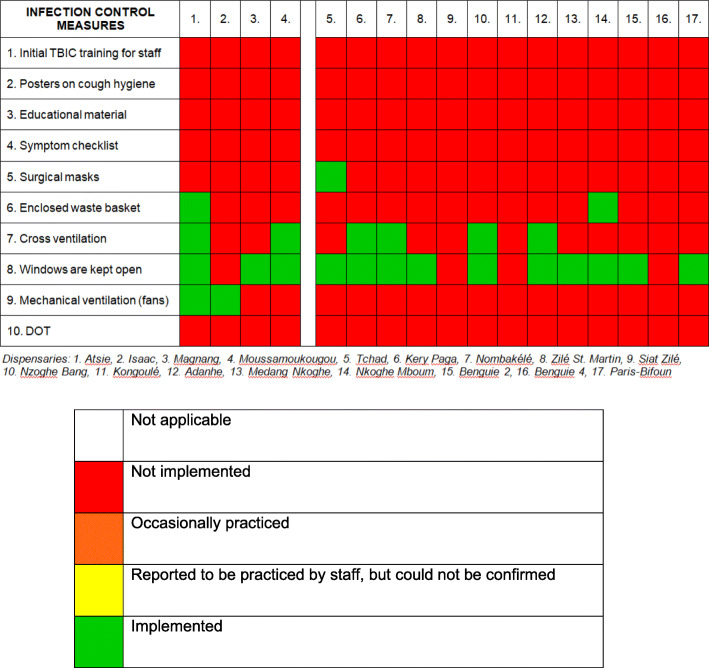
Infection control measures in dispensaries. Green colour indicated that the TBIC action was *Implemented*; orange colour indicated that the TBIC action was occasionally practiced, red colour indicated that the TBIC action was *Not implemented*

The implementation of TBIC measures in dispensaries is presented in Fig. 6. None of the staff had received training in TBIC. In addition, dispensaries were poorly equipped. None of them had posters on cough hygiene put up, or other educational material at hand. Only in one facility, surgical masks were available. In two dispensaries, there were closeable waste baskets.

### Comparison of primary care health facilities with referral hospitals

In general, the implementation of TBCI in primary health care facilities and referral hospitals is poor. However, compared to primary care health facilities, referrals hospital provide coughing patients with mask and applied the patients triage.

The primary healthcare facilities were found to have an advantage regarding environmental controls: There was cross-ventilation in 35,3 % of the dispensaries, and 76,5 % kept all windows open during working hours. Two urban dispensaries had additional mechanical ventilation.

## Discussion

We assessed TBIC measures in place compared with international recommendations, most notably from WHO. This was intended as establishing the baseline for the development of a national TBIC program that would be feasible to implement in Gabon. Only after specific guidelines are developed, their implementation process can be initiated and monitored by regular assessments. We chose to present our findings in colour-coded dashboards, which allows easy comparison when follow-up assessments are conducted, as it was done by Dokubo et al. in seven facilities in Nigeria [12].

Emerson at al. showed in their longitudinal study with pre- and post-interventional assessments in several out-patient HIV clinics in Zambia and Botswana that designing a plan specific for each facility and training of HCW can considerably improve the clinic performance within one year [13]. The national TBIC program will suggest plans according to the respective facility levels. In Gabon, the respective challenges are considered similar across facilities at the same level.

Furthermore, focus group discussions have been shown to be useful to identify obstacles in the implementation process. Inadequate facility structures, lack of human resources and managerial support, as well as stigmatisation of TB patients are frequently-mentioned obstacles [4, 14]. The focus group discussion provided additional details and explanations on why certain measures were not implemented correctly. We suggest to consider this in future qualitative studies assessing the application of TBIC. The tool was originally developed to assess an entire facility. In this study, different departments were observed separately. Therefore, some of the items did not apply to every department. For instance, there are no waiting areas on the hospital wards, as patients are directly hospitalized from the OPD or the ED, which is not optimal. Additionally, the tool did not include any means of verification for TBIC measures. The assessment period was not long enough to observe a TB patient attending every department, so measures that were reported to be practiced by hospital staff, could sometimes not be confirmed by observation. Therefore, additional observations were documented in the comments section and eventually included in the data analysis. Accordingly, a refined tool was developed after study completion, which can be used to obtain comparative follow-up data in future studies.

A limitation of the study was that in some departments, there was no presumed TB case during the assessment period. Thus, the management of presumed TB cases could not be observed directly and certain practices could therefore not be evaluated.

### Managerial controls

 The assessments provided evidence of the urgent need of national guidelines and specific facility plans as well as designated TBIC managers. Managerial controls are an indispensable pre-requisite for the implementation of TBIC measures and should therefore be given first priority[15].

### Administrative controls

Only a few administrative controls could be consistently observed in some departments. While none of the departments screened for cough, separation of coughing patients and provision of surgical masks were observed practice in both IM wards and the MT. However, these measures were not systematically implemented. Only two departments screened newly diagnosed HIV-positive patients for TB, and only one department (MT) tested its staff regularly. An optimized patient flow such as the one suggested by Verkuijl et al. [16] could facilitate the integration of administrative TBIC practices into the routine work and ensure their constant application.

### Environmental controls

Some environmental measures, such as windows on opposite walls or high ceilings, depend on the facility structure and can therefore not easily be changed. However, ensuring that windows are kept open and fans are being used to direct the airflow away from HCWs, is an easy, inexpensive and effective TBIC measure [17]. In our study, we observed that these measures were only constantly used in the isolation rooms of the MT. Particularly consultation rooms were not adequately ventilated; mostly due to the use of AC, thus posing a high risk for the consulting HCW.

### PPE

During the time of the study, adequate respirators such as N95 masks were available in two individual departments out of ten. In both cases, they were not provided by the facility; and only in one of the departments; regular use of these respirators was observed. These findings could indicate that there is a lack of awareness regarding the importance of PPE, and that a budget must be put in place for the provision of respirators to enable HCW to protect themselves from airborne infectious diseases.

Whereas appropriate respirators were unavailable in most departments, surgical masks were frequently used by HCW when dealing with a TB patient. As these masks do not protect individuals from becoming infected, facilities must ensure the provision of N95, or equivalent masks[18]. Furthermore, awareness must be raised, and HCW need to be trained to adequately use these respirators.

### Dispensaries

Dispensaries in Gabon are not authorized to treat TB patients. However, they must be able to identify presumed TB cases and refer them to a diagnostic and treatment center (CDT). With a median of 29 %, approximately a third of the patients was presenting to the dispensary, with cough as one of their main symptoms. Therefore, we assume that there is a considerable number of TB patients seeking healthcare in a dispensary. However, while reviewing the patient registers, we found that there was no documented case of presumed TB and referral to a CDT. Patients with fever and cough were usually diagnosed as ‘malaria with respiratory affection’. The majority of the dispensaries were run by assistant and/or auxiliary nurses, who were not adequately trained to identify patients who require referral for further TB diagnostics. These findings indicate that more attention must be paid to primary healthcare services in Gabon, as they play a crucial role in TB control. Ideally, they should also be integrated in TB treatment surveillance programs, which would be in line with the DOTS (Directly Observed Therapy - Short Course) strategy.

In general, regarding TBIC, the dispensaries had one strength: compared to the larger facilities, environmental controls in terms of natural ventilation were found to be generally more suitable for consultation rooms. Windows were kept open continuously during opening hours in most dispensaries, and there was cross-ventilation in six facilities out of twenty.

However, equipment was very basic. Mechanical ventilation was only available in two facilities out of twenty, due to the absence of electricity. Primary healthcare services play an important role in TB control and the provision of symptom checklists, educational material and surgical masks should be standard equipment.

In general, the implementation of TBIC was poor in all health facilities included in our survey. The reasons are manifold. Our finding is similar to those from previous reports from different African countries [4, 5, 11]. Across studies including our own, the main obstacles identified were limited resources such as inadequate space or consultation rooms, lack of staff or inadequately trained health workers, lack of protective gear and absence of regular monitoring and supervision of TBIC activities in the health care setting [4, 5, 11].The preliminary results of this study are of great utility since they served to provide clear data on the lack of infection control and especially on the need for a national TBIC guideline.

 In due course, infection control recommendations were added as an integral part into the national tuberculosis control guidelines. Furthermore, during supervision visits, the need to respect these control measures as recommended is encouraged by the national TB control program. CERMEL as research center, through its expertise [19–21], provided essential support for the implementation of this guideline, since its staffs contributed significantly to the drafting of this guideline. The major limitation of our study is the fact that we included health facilities from only one region. Future research should aim at assessing the situation countrywide, paving the way for the establishment of a routine continuous TBIC evaluation and safeguarding system, and should ideally include, alongside cost –effectiveness analyses,

## Conclusions

Our assessment findings indicate that in Moyen-Ogooué province, Gabon, TBIC implementation in healthcare settings is generally low, and has not yet been sufficiently addressed. Consequently, HCW are not sufficiently protected and therefore at enhanced risk of *M. tuberculosis* infection. Furthermore, the findings suggest that environmental measures could easily be optimized in all facilities, particularly in consultation rooms. There is an urgent need to develop national TBIC guidelines and training of health workers; and to provide respective appropriate resources to safeguard implementations, as well as to re-evaluate the evolving state of implementation regularly, in order to protect HCW as well as patients in these healthcare facilities from nosocomial TB infection.

## Supplementary information

Additional file 1 Study questionnaire as applied.

## Data Availability

The datasets used and/or analyzed during the current study are available from the corresponding author on reasonable request.

## Abbreviations

AC: air condition
BELE: Base d’épidémiologie
CDT: Centre de diagnostic et de traitement
CERMEL: Centre de Recherches Médicales de Lambaréné
CTA: centre de traitement ambulatoire (ambulatory HIV clinic)
DOTS: Directly Observed Therapy - Short Course
DRS: Regional Health Director
ER: emergency room
HCW: healthcare workers
HIV: human immunodeficiency virus
IM: internal medicine ward
MDR-TB: multi drug resistant tuberculosis
MT: MDR-TB treatment unit
OPD: outpatient department
PD: pediatric ward
PNLT: National Tuberculosis Programme
PPE: personal protective equipment
TB: tuberculosis
TBIC: tuberculosis infection control
UVGI: ultraviolet germicidal irradiation
WHO: World Health Organization
