# Supplementary material for: Tuberculosis infection control measures in healthcare facilities in Moyen-Ogooué Province, Gabon
Source: BMC Health Serv Res. 2021 Nov 5;21:1200. doi: 10.1186/s12913-021-07236-z (PMC8571857; doi:10.1186/s12913-021-07236-z)
Supplement: Supplementary file 1 — Study questionnaire as applied. [file 12913_2021_7236_MOESM1_ESM.docx]

**Supplementary Materials Legend**

**Supplementary Material 1 –** Modified CERMEL ICE Questionnaire

**Supplementary Material 1 –** Modified CERMEL-ICE Questionnaire

| **Facility**: _________________________________________________________________  **Department (if applicable**): _________________________________________________  **Period of assessment**: ____________________________________________________  **Assessed by:** ____________________________________________________________ |
| --- |

**Note:** If a practice is reported by the majority of staff and the head of department, but cannot be confirmed nor disproved by one of the means of verification, please note in the comment section.

**Abbreviations:** TB = tuberculosis, TBIC = tuberculosis infection control, HIV = human immunodeficiency virus

**1. MANAGERIAL CONTROLS**

|  | **2** | **1** | **0** | **NA** | **Means of verification** |
| --- | --- | --- | --- | --- | --- |
| 1. Is there a designated person and/or committee in charge of TBIC? | Yes |  | No | NA | Interview, organization chart |
| 2. Is there a written TBIC plan addressing TBIC and is it accessible onsite? | Yes | Yes, but not readily accessible | No | NA | TBIC plan |
| 3. Has the facility been assessed regarding TBIC in the last year? | Yes |  | No | NA | Assessment report |
| 4. Is there a budget for TBIC? | Yes |  | No | NA | Budget plan |

Score: _____/_____

Comments: __________________________________________________________________________________________________________________________________________________________________________________________________________________________________________________________________________________________________________________________________________________________________________________

Recommended actions:

__________________________________________________________________________________________________________________________________________________________________________________________________________________________________________________________________________________________________________________________________________________________________________________

**2. ADMINISTRATIVE CONTROLS**

|  | **2** | **1** | **0** | **NA** | **Means of verification** |
| --- | --- | --- | --- | --- | --- |
| 1. Are patients screened for cough as they enter the facility (triage)? | Yes | Sometimes | No | NA | Observation |
| 2. Are coughing patients/presumed TB cases prioritised? | Yes | Sometimes | No | NA | Observation |
| 3. Are coughing patients/presumed TB cases provided with surgical masks? | Yes | Sometimes | No | NA | Observation |
| 4. Are presumed TB cases/TB patients separated from other patients? | Yes | Sometimes | No | NA | Observation |
| 5. Were all newly diagnosed HIV+ patients screened for TB? | Yes | Some | No | NA | Patient files/HIV register |
| 6. Are posters about cough hygiene prominently displayed? | Yes | Yes, but not prominently | No | NA | Observation |
| 7. Are TB patients provided with educational material? | Yes | Occasionally | No | NA | Observation and/or patient interviews |
| 8. Are personnel tested for TB regularly? | Yes | Yes, but not regularly | No | NA | Register and/or interviews |
| 9. Do employees have access to confidential HIV counseling and testing onsite? | Yes |  | No | NA | Register and/or interviews |
| 10. Is isoniazid preventive therapy available for HIV positive staff? | Yes |  | No | NA | Register and/or interviews |
| 11. Is initial training provided for new staff members about TBIC practices? | Yes | Some staff | No | NA | Training Log |
| 12. Are there separate and ventilated facilities for sputum collection? | Yes | Yes, but not ventilated | No | NA | Observation |

Score: _____/_____

Comments: __________________________________________________________________________________________________________________________________________________________________________________________________________________________________________________________________________________________________________________________________________________________________________________

Recommended actions:

____________________________________________________________________________________________________________________________________________________________________________________________________________________________________________________________________________________________________________________________________________________________________________________________________________________________________________________________

**3. ENVIRONMENTAL CONTROLS**

|  | **2** | **1** | **0** | **NA** | **Means of verification** |
| --- | --- | --- | --- | --- | --- |
| 1. Is there cross ventilation in waiting area/isolation rooms? | Yes | Sometimes | No | NA | Observation |
| 2. Are there high ceilings (> 3m) in the facility? | Yes | Sometimes | No | NA | Observation |
| 3. Are fans used in the waiting hall/isolation rooms? | Yes | Sometimes | No | NA | Observation |
| 4. Are windows kept open and are fans being used in the consultation rooms? | Yes | One of both | No | NA | Observation |

Score: ____/____

Comments: __________________________________________________________________________________________________________________________________________________________________________________________________________________________________________________________________________________________________________________________________________________________________________________

Recommended actions:

____________________________________________________________________________________________________________________________________________________________________________________________________________________________________________________________________________________________________________________________________________________________________________________________________________________________________________________________

**4. PERSONAL PROTECTIVE EQUIPMENT**

|  | **2** | **1** | **0** | **NA** | **Means of verification** |
| --- | --- | --- | --- | --- | --- |
| 1. Are N95 respirators or equivalent masks available? | Yes | Yes, but not consistently | No | NA | Observation and stock records |
| 2. Were N95 respirators or equivalent masks used in risk areas this month? | Yes | Sometimes | No | NA | Observation |

Score: ____/____

Comments: __________________________________________________________________________________________________________________________________________________________________________________________________________________________________________________________________________________________________________________________________________________________________________________

Recommended actions:

____________________________________________________________________________________________________________________________________________________________________________________________________________________________________________________________________________________________________________________________________________________________________________________________________________________________________________________________

**5. ADDITIONAL INFORMATION**

Is a TB register available? 🞎 Yes 🞎 No

If yes, how many TB cases (all types) where registered in the past calendar year? ____________________________________________________________________

Are sputum samples analysed onsite? 🞎 Yes 🞎 No

If no, where are sputum samples sent?

____________________________________________________________________

If yes, what analyses are available?

________________________________________________________________________________________________________________________________________________________________________________________________________________________________________________________________________________

Where and how is sputum sample collection performed?

______________________________________________________________________________________________________________________________________________________________________________________________________________________________

Are TB patients treated onsite? 🞎 Yes 🞎 No

If no, where are TB patients referred to?

____________________________________________________________________

If yes, describe how TB patients are followed up:

________________________________________________________________________________________________________________________________________________________________________________________________________________________________________________________________________________________________________________________________________________________________________________________________________________________
